# A Multidisciplinary Review of Phytoremediation Strategies for Heavy Metal-Contaminated African Soils: From Geochemical Assessment to Genetic Enhancement

**DOI:** 10.3390/jox16030118

**Published:** 2026-06-22

**Authors:** Fatouma Mohamed Abdoul-Latif, Rohit Kumar, Talal Mohamed, Ali Merito, N Chinmaya Kumar, Ibrahim Houmed Aboubaker, Pannaga Pavan Jutur

**Affiliations:** 1 Medicinal Research Institute, Center for Studies and Research of Djibouti (CERD), Djibouti BP 486, Djibouti; 20karim14@gmail.com (T.M.); alimerito@gmail.com (A.M.); ibrahimhoumed@yahoo.fr (I.H.A.); 2 Department of Biotechnology, Sri Ramaswamy Memorial Institute of Science and Technology, Delhi-NCR, Sonipat 131029, India; kumarrohitt121998@gmail.com; 3 Ganesh Scientific Research Foundation, Kirti Nagar, Delhi 110015, India; 4 Department of Botany, Government Autonomous College, Angul 759122, India; nchinmayakumar3@gmail.com; 5 International Centre for Genetic Engineering and Bio-Technology, Aruna Asaf Ali Marg, New Delhi 110067, India; pavan.jutur@icgeb.org

**Keywords:** heavy metals, African soils, phytoremediation, hyperaccumulators, speciation, machine learning, integrated framework

## Abstract

African soils face increasing levels of metal pollution due to industrialization, artisanal mining activities, improper waste management, and enhanced agricultural productivity. However, unlike many organic pollutants, heavy metals do not degrade naturally and therefore persist in environmental systems for prolonged periods. Heavy metals accumulate over many decades in the soil and bioaccumulate through the food chain causing severe health complications such as cancer, kidney problems, and neurological impairment. This paper reviews the current literature on the origin, prevalence, and behavior of the main pollutants Pb, Cd, Cr, As, Hg, and Cu. The major phytoremediation methods including phytoextraction, rhizofiltration, phytostabilization, and phytovolatilization are highlighted alongside in planta screening methods for hyperaccumulating plants including *Berkheya coddii* (Ni) and *Haumaniastrum robertii* (Co). The paper evaluates various enhancement techniques such as the use of chelators, Rhizobium inoculations, and genetic modifications. The significance of these approaches in tropical and subtropical climates is discussed. The paper suggests a holistic framework involving empirical kinetic modeling, geospatial machine learning (random forest, kriging), and molecular omics in prediction modeling. Major hurdles in such predictions include lack of field-based verification of the models, biotechnology safety of genetically modified (GM) organisms, and inadequate regulations. Future perspectives emphasize community-driven phytomining, biomass recycling, and resilient phytoremediation solutions.

## 1. Introduction

The heavy metal pollution of the soil is considered among the most complex and long-lasting impacts produced by humans on nature throughout the time of Anthropocene. However, in Africa, the issue becomes more fascinating because of a unique set of conditions [1]. Firstly, Africa contains some of the largest global reserves of base and precious metals. Secondly, severe soil contamination has emerged due to rapid urbanization, industrial expansion, and poorly regulated artisanal and small-scale mining (ASM), especially in regions with limited environmental governance and remediation infrastructure. The countries of Africa do not have enough economic resources to implement remedial actions compared to Europe or America [2].

Metals having densities higher than 5 g/cm^3^, and which are poisonous and can be ecotoxic are termed heavy metals. These include Pb, Cd, Cr, As, Hg, Ni, Cu, and Zn [3]. Even though some may be essential micronutrients (such as Ni, Cu, and Zn), in contrast, noble metals such as gold and silver are generally less toxic than most other heavy metals, although excessive exposure or accumulation may still produce adverse biological effects. The FAO/WHO has even raised concerns regarding the high amounts of metals found in the crops grown in Africa, particularly around peri-urban regions such as Lagos (Nigeria), Nairobi (Kenya), and Accra (Ghana) [4].

Phytoremediation technology, which involves the use of plants along with their microbial symbionts in the removal or stabilization of contaminants in the environment, is a particularly attractive option in the case of implementation in Africa [5]. Phytoremediation processes are site-specific, driven by solar energy, maintain soil structure, and can be coupled with agricultural activities (phytomanagement). Despite the extensive research and developments in the last twenty years, however, very few practical phytoremediation projects have emerged in Africa [6]. The most crucial weaknesses are a lack of screening of native African plant species having hyperaccumulator capacity; field-scale phytoremediation applications remain limited across many African regions because of inadequate long-term validation studies and infrastructure constraints; and the failure to bridge laboratory research to field applications [7].

This review provides a multidisciplinary perspective on heavy metal contamination and phytoremediation strategies by integrating soil science, environmental geochemistry, plant biotechnology, molecular remediation approaches, and predictive modeling tools. Unlike earlier generalized reviews, this integrated framework incorporates broader environmental perspectives while still highlighting contamination scenarios and remediation challenges relevant to African ecosystems. The manuscript further emphasizes mining-associated contamination, agricultural intensification, industrial emissions, climatic variability, and socioeconomic limitations that influence heavy metal mobility and remediation feasibility in developing regions. In addition, emerging roles of machine learning, geospatial analysis, microbial-assisted phytoremediation, and genetic enhancement technologies are discussed within an integrated environmental remediation framework.

## 2. Methodology

A comprehensive literature search was conducted using major scientific databases, including Scopus, Web of Science, PubMed, Google Scholar, and ScienceDirect. Keywords such as “heavy metal contamination in African soils,” “phytoremediation,” “hyperaccumulator plants,” “phytomining,” “machine learning in environmental remediation,” and “African soil pollution” were used individually and in combination. Only peer-reviewed articles published in English were included. Studies lacking sufficient methodological detail or unrelated to African environmental contexts were excluded. The selected literature was critically evaluated based on relevance, methodological quality, regional applicability, and contribution to phytoremediation strategies in Africa.

## 3. Sources and Distribution of Heavy Metals in African Soils

### 3.1. Natural and Anthropogenic Sources

Heavy metals in African soils can originate from the weathering of parent rocks, although the degree of accumulation varies depending on differential weathering processes, rock mineralogy, and the geochemical behavior of individual metals. The old cratons of West Africa, Congo, Kalahari, and Tanzania have varying levels of heavy metals, with the Copperbelt of Zambia and DRC accounting for 10% of the global reserves of copper, while the Bushveld Igneous Complex of South Africa is rich in platinum group metals and chromium [2]. The volcanics of the East African Rift province are responsible for increasing the concentration of nickel, chromium, and cobalt.

In contrast, anthropogenic sources dominate in certain areas. The major source of mercury and lead pollution in sub-Saharan Africa is small and artisanal gold mining. Mercury vapor is emitted by burning amalgam, which is used by gold miners for amalgamation [8]. Some 1000 metric tons of mercury are released annually as a result of Artisanal and small-scale gold mining (ASGM) activities in the region. ASGM is considered one of the major anthropogenic sources of heavy metal contamination in several African regions due to the extensive use of mercury during gold extraction processes. The process of recycling battery acid in urban informal settlements (for example, Owino Uhuru, Kenya; Dakar, Senegal) is responsible for the highest levels of soil lead pollution. The environmental impact of ASGM is commonly reported in terms of annual mercury emissions or releases (metric tonnes per year), which quantify the total mass discharged into the environment, whereas concentrations of heavy metals in contaminated soils are typically expressed in mg/kg, representing contaminant accumulation per unit mass of soil. Agricultural pollutants include phosphate fertilizers (cadmium), sewage sludge (zinc, copper, and lead), livestock manure, and pesticide applications, which can contribute substantial copper and arsenic contamination to soils. Industrial pollutants include tannery wastes (chromium), dye factory wastewaters (chromium and copper), and electroplating wastes [9].

### 3.2. Major Contaminants of Concern

Major heavy metal contaminants found in soils include lead (Pb), cadmium (Cd), chromium (Cr), mercury (Hg), arsenic (As), nickel (Ni), copper (Cu), and zinc (Zn). These contaminants originate from mining activities, industrial discharge, agricultural practices, electroplating industries, wastewater irrigation, and improper waste disposal. Prolonged accumulation of these metals in soils negatively affects soil fertility, microbial activity, crop productivity, and ecosystem stability.

(a) Pb: Most widespread. Neurodevelopmental toxicity in children [10]. Average Pb level in African urban gardens: 150–600 mg/kg (versus background of 10–30 mg/kg). Cd exhibits high mobility and bioaccumulation potential. Phosphate fertilization has increased cadmium concentrations in East African soils (Rift Valley, Kenya) to approximately 2–8 mg/kg, exceeding the European Union agricultural soil threshold values for cadmium, with observed values ranging between 1–3 mg/kg depending on soil characteristics (1.5 mg/kg) [2].
(b) As: A toxic metalloid commonly associated with mining and industrial contamination. Arsenic contamination in Africa arises from both natural and anthropogenic sources, including geogenic mobilization in volcanic regions such as Ethiopia and Tanzania, as well as the weathering and dispersal of arsenic-rich mine tailings associated with mining activities in countries such as Zimbabwe and South Africa. Associated with dermatological and carcinogenic diseases [11].
(c) Hg: Mercury contamination is primarily associated with ASGM. Unlike most other heavy metals, inorganic mercury can be transformed by microorganisms into methylmercury, a highly toxic form that readily bioaccumulates and biomagnifies through aquatic food chains. Enhanced methylmercury production in wetlands and other anoxic environments poses a significant risk of neurological damage to both wildlife and human populations through dietary exposure, particularly via fish consumption [12].
(d) Cr: Hexavalent chromium (Cr(VI)) through tanneries (Ethiopia, Nigeria) and chromium ore mining (South Africa). Cr (VI) is classified as a Group 1 carcinogen [13].
(e) Cu: From excess mining (Zambia, DRC) and fungicides. Cu may exhibit phytotoxic effects at elevated bioavailable soil concentrations (typically >100 mg/kg). In addition, its well-established fungitoxic activity underpins the extensive use of copper-based formulations in agriculture for the control of fungal diseases [14]. Although several contamination pathways summarized in Table 1 are globally recognized, these sources remain highly relevant across African environmental systems due to extensive mining activities, rapid urbanization, industrial emissions, agricultural intensification, and insufficient waste-management infrastructure.

### 3.3. Regional Distribution and Hotspots

Heavy metal pollution in African soils displays distinct regional trends associated with their industrial and mining practices [21]. The Niger Delta area of Nigeria, for instance, exhibits a high degree of contamination of its soils by heavy metals such as Pb, Cr, and Ni due to oil exploration activities as well as informal metal recycling in the area. Artisanal gold mining in the countries of Burkina Faso, Mali, and Ghana is the source of considerable Hg contamination in the soils, which contain up to 50 mg/kg Hg in comparison with the background value of 0.05 mg/kg [22]. In the East African part of the continent, Hg and As contamination occur in Lake Victoria Goldfields that cover Tanzania, Kenya, and Uganda, while soil contamination by hexavalent chromium (Cr VI) up to the level of 4000 mg/kg occurs in the Nairobi Athi River area of Kenya, where tannery effluents are discharged [23]. Major contamination hotspots include the Zambian Copperbelt and Lubumbashi region of the Democratic Republic of Congo, where prolonged Cu-Co mining activities have resulted in elevated concentrations of copper, cobalt, cadmium, and lead in surrounding soils. Similarly, artisanal gold-mining regions in Ghana, Nigeria, and Tanzania are associated with mercury contamination, while peri-urban agricultural systems in Kenya exhibit elevated cadmium and pesticide-associated metalloid accumulation due to intensive fertilizer and agrochemical use. The Zambia–DRC Copperbelt region in southern Africa is a globally significant Cu–Co mining province. Mining and smelting activities have resulted in severe soil contamination, with reported Cu concentrations reaching several thousand mg/kg and Co concentrations exceeding 500 mg/kg in areas proximal to industrial operations [24]. Similarly, soils in the Highveld area of South Africa (Johannesburg-Pretoria) demonstrate high Pb pollution due to past leaded gasoline consumption and mining activities, and Zimbabwe’s Great Dyke region displays significant Ni and Cr areas of elevated concentration. In northern Africa, particularly in the phosphate-mining regions of Settat and Khouribga (Morocco), agricultural soils may accumulate Cd and U through the long-term application of phosphate fertilizers derived from phosphate rock, in which both elements occur naturally [25]. Additional U inputs may also originate from atmospheric deposition associated with coal combustion [26]. However, in Egypt, the soils of the Nile Delta receive Pb, Cd, and Ni through industrial and residential wastewater irrigation.

Mining and smelting activities in the Zambian Copperbelt and Lubumbashi region (DRC) have resulted in elevated concentrations of Cu, Co, Pb, and Cd in surrounding agricultural soils and water bodies. Similarly, ASGM activities in Ghana and Tanzania are major sources of mercury contamination due to the widespread use of poorly regulated mercury amalgamation practices.

Kabwe, Zambia, is recognized as one of the world’s most severely lead-contaminated mining regions, where historical mining and smelting activities have resulted in extensive Pb accumulation in soils, posing significant risks to human health through dust inhalation, soil ingestion, and the consumption of contaminated food crops. Although lead generally exhibits low solubility and limited mobility in most soils, elevated concentrations may persist for decades due to its strong association with soil particles. In peri-urban agricultural systems surrounding Nairobi, Kenya, wastewater irrigation and industrial effluents have contributed to the accumulation of Cr, Cd, and Pb in cultivated soils, raising concerns regarding food safety and chronic human exposure [21,23,27,28].

## 4. Environmental and Human Health Impacts

### 4.1. Environmental Fate and Transport

The bioavailability of heavy metals in soils of Africa depends on the soil pH, organic matter content, cation exchange capacity, and mineral composition. In the case of tropical soils known as Ferralsols with low pH levels (4.5–5.5), cadmium and zinc are highly soluble and can leach into groundwater [27]. Lead and chromium (III) show high affinity for adsorption to iron and manganese oxides. The reducing environment found in irrigated rice paddies (such as in Nigeria and Madagascar) converts chromium (VI) to chromium (III) and also methylates mercury [29].

Chronic exposure to heavy metals and metalloids through contaminated food, drinking water, and inhalation pathways is associated with severe health consequences, including neurotoxicity, nephrotoxicity, developmental disorders, endocrine disruption, and carcinogenicity, with arsenic (As) recognized as a well-established human carcinogen. Lead exposure has been strongly associated with neurological impairment in children, whereas cadmium accumulation may result in renal dysfunction and skeletal abnormalities.

Mercury contamination from artisanal and small-scale gold mining poses serious ecological and public health risks, as methylmercury bioaccumulates in aquatic organisms and biomagnifies through food chains, increasing human exposure through the consumption of contaminated fish. Similarly, hexavalent chromium exposure has been associated with respiratory toxicity, mutagenicity, and increased cancer risk [12,13,16,19,28,30].

**Figure 1:**
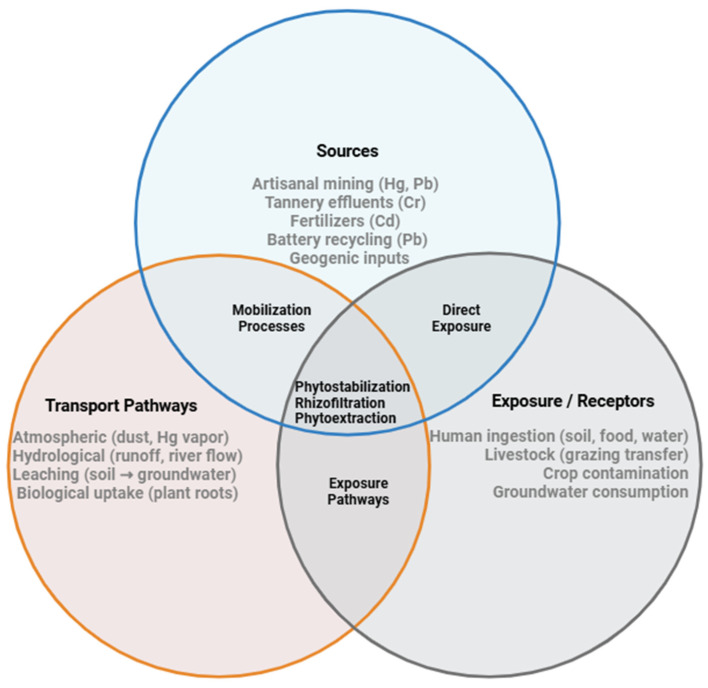
Interrelationship between sources, transport pathways, and exposure/receptors of heavy metal contamination in African soils. The *Sources* circle includes both anthropogenic (artisanal mining, tannery effluents, fertilizers, battery recycling) and geogenic inputs. The *Transport Pathways* circle represents key mechanisms of metal mobilization, including atmospheric dispersion, hydrological runoff, leaching into groundwater, and biological uptake by plants. The *Exposure/Receptors* circle highlights human and ecological exposure routes such as soil, food, and water ingestion, livestock transfer, crop contamination, and groundwater consumption. Overlapping regions indicate critical processes: *Sources–Transport* reflects mobilization processes; *Transport–Exposure* denotes exposure pathways; and *Sources–Exposure* represents direct exposure scenarios. The central intersection emphasizes the source–transport receptor nexus, which serves as the primary target for phytoremediation strategies, including phytostabilization, rhizofiltration, and phytoextraction.

The colloid-assisted transport process is an important yet underexplored mechanism in African Oxisols, where nanometric hematite and goethite particles assist in the transportation of bound metals to the drainage system. Smelter emissions serve as atmospheric contaminants, and lead from the Kabwe mine in Zambia has been found in soils 50 km downwind [30]. Therefore, additionally interrelationship between sources, exposure/receptors and transport pathways is shown in Figure 1.

### 4.2. Human Health Risks

There are three major routes through which exposure can occur: direct soil ingestion (children with pica), consumption of contaminated food and drinks, and inhalation of resuspended dust. The intake route is responsible for 70–90% exposure for non-occupational groups [28].

Risk assessments using the Hazard Quotient (HQ) model and Cancer Risk (CR) developed by US EPA have been used across various African countries. HQs for cadmium from contaminated leafy vegetables (Amaranthus and Celosia) in Nigeria are more than 1, showing the non-carcinogenic nature [31]. The average concentration of blood lead among children in Kabwe, Zambia is between 30–50 μg/dL (action level is 5 μg/dL), thus causing irreversible decrease in IQ. There is an association between chronic arsenic exposure and hyperkeratosis/bladder cancer in Ethiopian Rift valley area [32]. The socioeconomic aspects increase the risk where poverty means living on contaminated lands, lacking clean water for washing food products, and disposal of harvested contaminated plant biomass from polluted wetlands are essential to prevent secondary environmental contamination.

Although HQ and CR models were originally developed by the United States Environmental Protection Agency (US EPA), these frameworks have been widely applied in African environmental-health studies investigating contamination risks associated with mining regions, agricultural soils, and urban-industrial ecosystems [33,34].

### 4.3. Socioeconomic Implications

Soil pollution degrades soil health and compromises the quality and safety of agricultural produce by promoting the accumulation of toxic contaminants in crops, even when visible effects on crop yield are limited. On the Copperbelt, people have left farming lands because of copper’s harmful effect on plant growth (Cu phytotoxicity). Metal-induced diseases and illnesses increase the medical expenses in health care budgets that are already struggling. Finally, residents of heavily contaminated “toxic villages,” such as Owino Uhuru in Kenya, frequently face social stigma, economic marginalization, reduced property values, and psychological stress, exacerbating the health and socioeconomic burdens associated with environmental pollution. Thus, remediating soil pollution is important both environmentally and socially [35].

## 5. Analytical Approaches for Soil Characterization

Reliable remediation requires accurate characterization. African laboratories face challenges of equipment cost, consumable supply chains, and quality assurance. This section outlines tiered approaches [36].

### 5.1. Sampling and Physicochemical Properties

Sampling strategy should take into consideration the effects of spatial heterogeneity. In cases where contamination levels exceed regulatory thresholds, integrated remediation strategies should be adopted to improve treatment efficiency. For regional contamination assessments, systematic grid-based sampling designs are commonly applied to improve spatial representation of soil contamination patterns. However, optimal grid spacing depends on factors such as pollution-source distribution, geological variability, and contamination heterogeneity. In moderately heterogeneous environments, a spacing of approximately 50 m × 50 m may be suitable, while composite samples collected from 0–20 cm and 20–40 cm depths are frequently used for topsoil and subsoil characterization, respectively [37]. The physicochemical characteristics of the soil sample that should be analyzed include the pH (ratio 1:2.5 water/0.01 M CaCl_2_), electrical conductivity, organic carbon content (Walkley-Black method), cation exchange capacity (0.1 M NH_4_OAc), particle size distribution (hydrometer analysis), and the contents of iron and manganese oxides Fe/Mn/Al oxides (dithionite citrate bicarbonate) present in the soil matrix [38].

### 5.2. Heavy Metal Quantification Techniques

Field Screening: X-ray fluorescence (XRF) analysis has become inexpensive (USD 15,000 to 25,000) and widely applied in mining regions of Africa. The detection limits for Pb, Cu, Zn, As are around 10–20 mg/kg, which is adequate for hot spots delineation [39]. However, portable X-ray fluorescence (pXRF) cannot analyze mercury and metal bioavailability and chemical speciation. Total Digestion: The samples may be digested using aqua regia (3:1 HCl:HNO_3_) or other HF/HClO_4_ digestion with subsequent analysis by ICP-OES or ICP-MS. For low levels of As and Cd analyses, ICP-MS should be applied. FAAS is still widespread owing to its low cost [40]. Quality Assurance: Certified reference materials such as NIST SRM 2710a (Montana I Soil) have to be analyzed together with every 20 samples. Inter-laboratory testing in Africa (e.g., AfriSA) has improved data quality.

### 5.3. Speciation and Bioavailability

Sequential metal extraction protocols (Tessier, BCR) have operational definitions for metal fractions: exchangeable, reducible (Fe-Mn oxides), oxidizable (organic), and residual. The exchangeable fraction is related to the rate of plant uptake [41]. Routine risk assessment calls for one extraction using 0.01 M CaCl_2_ or DTPA (diethylenetriaminepentaacetic acid).

Diffusive gradients in thin films (DGT) is a better method for estimating metal fluxes; however, DGT technology requires specialized skills that are still rare in Africa [42].

X-ray Absorption Spectroscopy (XAS) offers genuine molecular speciation (e.g., Cr (III) versus Cr (VI)); however, synchrotron facilities are scarce. Alternatively, portable colorimetric spot tests (e.g., diphenylcarbazide for Cr (VI)) can be applied in the field also shown in Table 2 [33].

## 6. Phytoremediation Strategies and Plant Screening

### 6.1. Mechanisms of Phytoremediation

Four major processes are utilized. Phytoextraction: Hyperaccumulator plants concentrate metals through their roots and transfer the metals to harvestable shoots. Efficient phytoextraction depends not only on metal uptake capacity but also on biomass productivity, root architecture, climatic adaptability, and translocation efficiency. Hyperaccumulator plants are generally considered suitable for phytoextraction when shoot metal concentrations exceed 1000 mg/kg for Ni, Co, Cu, and Pb, or 10,000 mg/kg for Zn and Mn under natural growth conditions. Particularly suitable for the remediation of Ni, Co, Zn, and Cd contamination. Phytoextraction is most effective when plant species combine high biomass production with hyperaccumulation capacity. Hyperaccumulator plants are generally defined as those capable of accumulating metal concentrations exceeding approximately 0.1% (1000 mg/kg dry weight) for Ni, Co, Cu, and Pb, and greater than 1% (10,000 mg/kg dry weight) for Zn and Mn in their aboveground tissues [46]. Phytostabilization: Tolerance plants trap metals in the rhizosphere through precipitation, adsorption, and reduction reactions [47]. This mechanism is particularly important in mining-affected African soils where complete metal removal may not be feasible due to economic or environmental limitations. Root exudates, microbial interactions, and soil amendments significantly influence metal immobilization efficiency and long-term stabilization. Although phytovolatilization reduces soil metal concentrations, concerns remain regarding atmospheric redistribution and ecological safety, particularly for mercury-contaminated environments. Phytostabilization strategies involving tolerant plant species can significantly reduce the mobility and bioavailability of Pb, Cr, and As through rhizosphere adsorption, precipitation, and immobilization mechanisms [48]. Appropriate for Pb, Cr, and As. No removal occurs, but metal mobility and availability are decreased. Rhizofiltration: Plants absorb metals from wastewater through aquatic or semiaquatic plants like *Eichhornia crassipes*. Rhizofiltration has shown considerable potential for treating industrial wastewater contaminated with Cd, Cr, Pb, and Hg due to the extensive root surface area and rapid biomass production of aquatic plants. Efficient in cleaning industrial effluent prior to discharge. Phytovolatilization: Plants absorb metals such as Hg and metalloid Se and volatilize them as mercury vapor (Hg^0^) and dimethyl selenide to the atmosphere [49].

### 6.2. In-Planta Screening Approaches

Plant species must be systematically screened under African environmental conditions to identify suitable phytoremediation candidates, since the wide range of climatic, geological, and soil conditions across the continent strongly influences plant adaptation and metal-accumulation efficiency. This process typically begins with the collection of soil and plant samples from contaminated sites, followed by thorough cleaning and acid digestion of plant tissues, including roots, shoots, and leaves, to quantify metal concentrations [50]. Key evaluation metrics include the Bioconcentration Factor (BCF), calculated as the ratio of metal concentration in the plant to that in the soil, and the Translocation Factor (TF), defined as the ratio of metal concentration in the shoot to that in the root. Plants exhibiting high bioconcentration factor (BCF > 1) and translocation factor (TF > 1) values are generally regarded as promising candidates for phytoextraction, as they demonstrate efficient metal uptake from soil and effective translocation from roots to aboveground tissues. Plant species are considered potential hyperaccumulators when both BCF and TF values exceed 1, along with meeting specific shoot concentration thresholds for example, greater than 1000 mg/kg for nickel and 100 mg/kg for cadmium. Although plants with BCF and TF values greater than 1 are generally considered capable of metal accumulation and translocation, substantially higher values are typically preferred for efficient and economically viable phytoextraction under field conditions [51]. To ensure reliability and applicability, screening should be conducted under both natural field conditions and controlled hydroponic systems. Screening under both field and hydroponic conditions is important because environmental parameters such as soil pH, organic matter content, salinity, microbial activity, and climatic variability strongly influence metal bioavailability and plant accumulation efficiency.

### 6.3. Native African Hyperaccumulators

The diversity of ultramafic soils, mining regions, and metalliferous ecosystems across Africa has contributed to the evolution of several unique hyperaccumulator species with exceptional metal tolerance and accumulation capacities. Sub-Saharan African phytoremediation programs increasingly emphasize indigenous hyperaccumulator species due to their superior climatic adaptability, lower maintenance requirements, and ecological compatibility under local environmental conditions [24,46,52]. Africa possesses some of the most interesting hyperaccumulators because of ultramafic soils and Co-Cu province: Nickel: *Berkheya coddii* (Asteraceae), a plant from South Africa, is a hyperaccumulator of Ni (up to 38,000 mg Ni/kg), which makes it the most effective dicot accumulator of this metal. The exceptional Ni accumulation capacity and high shoot biomass of *Berkheya coddii* make it one of the most promising species for large-scale phytomining and phytoextraction applications under semi-arid environmental conditions. Senecio coronatus is another promising hyperaccumulator species, with the ability to bioaccumulate Ni in its aboveground tissues at concentrations of up to approximately 5000 mg/kg dry weight. Cobalt and Copper: *Haumaniastrum robertii* (Lamiaceae) from the Copperbelt of the DRC is a hyperaccumulator of both Co (up to 3000 mg/kg) and Cu (over 2000 mg/kg). *Gladiolus kilimanus* hyperaccumulates both metals as well. Cadmium: *Bidens pilosa*, an abundant weed, demonstrates significant Cd accumulation (200–400 mg/kg), which, combined with its substantial biomass, allows it to be used for phytoextraction after chelate addition. In addition to its Cd accumulation potential, the rapid growth rate and wide ecological adaptability of *Bidens pilosa* enhance its suitability for field-scale remediation programs. Arsenic: *Pteris vittata* (Chinese brake fern) is not a native species, but it has naturalized in parts of West Africa; it is an arsenic hyperaccumulator (up to 5000 mg/kg) [34]. The search for native Pteris species continues. Lead: There is no hyperaccumulator of Pb.

Despite the presence of several hyperaccumulator species, further exploration of African-native metallophytes remains necessary to identify plants with improved biomass production, climatic adaptability, and multi-metal remediation capabilities.

### 6.4. Enhancement Strategies

Natural hyperaccumulators tend to be slow-growing. One of the major limitations of phytoremediation is the trade-off between metal accumulation efficiency and biomass productivity, as many hyperaccumulator species exhibit relatively slow growth and low biomass under field conditions. The goal of enhancement technologies is either higher metal bioaccumulation or better biomass. Another approach to improve phytoextraction efficiency involves the use of chelating agents. The addition of EDTA or NTA can enhance the mobilization and plant uptake of Pb, Cu, and Cd by increasing their bioavailability in soil. However, these synthetic chelates may also increase the risk of metal leaching into groundwater. Consequently, biodegradable alternatives such as EDDS are generally preferred because they offer improved environmental compatibility. Chelating agents increase metal solubility and root uptake; however, excessive application may increase the risk of metal leaching into groundwater systems, particularly in sandy or low-organic-matter soils. In South African experiments, *Brassica juncea* and EDDS improved Pb extraction five times. Inoculation of rhizobacteria: Plant growth-promoting rhizobacteria (PGPR), such as “*Pseudomonas*” spp. or “*Bacillus*” spp. produce siderophores, ACC deaminase, and IAA [53]. Chelating agents increase metal solubility and root uptake; however, excessive application may increase the risk of metal leaching into groundwater systems, particularly in sandy or low-organic-matter soils. “*Bacillus subtilis*” inoculation in Nigeria experiments increased Cd extraction by “Amaranthus hybridus” by 60%. Arbuscular mycorrhizal fungi (AMF): AMF improve phosphorus and metal extraction. On the other hand, AMF can immobilize metals in the root system. Isolation of African-native strains of AMF, such as “*Rhizophagus irregularis*,” from mine tailings has been studied [54]. AMF-mediated phytoremediation may either enhance phytoextraction or promote phytostabilization depending on fungal species, metal type, soil chemistry, and host-plant interactions. Soil amendments such as agricultural lime (CaCO_3_) can increase soil pH and thereby reduce the bioavailability and mobility of Pb and Cd through chemical immobilization. When combined with vegetation establishment, liming can also support phytostabilization by improving plant growth and enhancing long-term contaminant stabilization. Conversely, elemental sulfur acidifies soil, increasing phytoextraction of Cd and Zn. Organic amendments, biochar, compost, and lime applications are increasingly used to modify soil pH, improve microbial activity, reduce metal toxicity, and enhance long-term remediation efficiency.

### 6.5. Biomass Management and Phytomining

Management of harvested metal-rich biomass remains one of the most critical challenges limiting large-scale implementation of phytoremediation technologies. Harvested contaminated biomass requires environmentally safe disposal or valorization strategies to avoid secondary contamination. Biomass incineration, biochar production, anaerobic digestion, and phytomining have emerged as promising management approaches [34,51,53]. Disposal of harvested metal-rich biomass represents another issue. These include incineration and smelting: High-temperature incineration extracts metals from ash. This approach substantially reduces biomass volume and facilitates metal recovery, although operational costs and energy requirements may limit large-scale implementation.

Although energy-intensive, it may be economical for extracting Ni and Co (“phytomining”). The ashes from “*Berkheya coddii*” may have up to 10–20% Ni [55]. Pyrolysis into biochar, it produces stable carbon that can be used as soil amendment; however, metal leachability must be assessed. Anaerobic digestion: Applicable only when metal concentration is low; metals are concentrated in digestate [56]. Biochar production may additionally improve soil quality and carbon sequestration; however, careful assessment of residual metal mobility and leachability is required before agricultural application. Compaction and disposal in landfills when metal levels are below hazardous waste criteria. Phytomining of metals by plants (“mining”) has been attempted in South Africa for Ni. Nickel phytomining systems may provide economic recovery opportunities in metal-rich ultramafic soils while simultaneously reducing contamination levels. The economic feasibility of phytomining depends on both biomass productivity and the concentration of Ni accumulated in the harvested shoots. For example, a hyperaccumulator crop producing approximately 10 t ha^−1^ of dry biomass containing around 1% Ni (10,000 mg kg^−1^ dry weight) would yield nearly 100 kg Ni ha^−1^ yr^−1^. At current Ni market prices (approximately USD 15,000–20,000 per tonne), this could generate a gross revenue of about USD 1500–2000 ha^−1^ yr^−1^, indicating the potential economic viability of agromining systems under suitable conditions [57]. The economic feasibility of phytomining depends on biomass yield, metal concentration, harvesting costs, processing efficiency, and prevailing market prices of target metals. Collectively, these integrated phytoremediation strategies demonstrate significant potential for sustainable remediation of heavy metal–contaminated soils, particularly when combined with microbial technologies, geospatial monitoring tools, biomass valorization approaches, and regionally adapted plant species.

## 7. Modelling and Predictive Tools

### 7.1. Empirical and Kinetic Models

Empirical models are widely used to estimate the relationship between metal bioavailability and key soil physicochemical properties. Regression-based approaches, including Freundlich-type empirical models, provide a first-order approximation of plant metal uptake by correlating metal concentrations in plants with soil characteristics. A commonly used model is expressed as: [\log(C_p) = a + b, \log(C_s) + c, (\mathrm{pH}) + d, \log(\mathrm{OM})] where (C_p) is the metal concentration in plant tissues, (C_s) is the total metal concentration in the soil, pH represents soil acidity or alkalinity, OM denotes soil organic matter content, and (a), (b), (c), and (d) are empirically derived regression coefficients. Logarithmic transformation is commonly applied to linearize nonlinear relationships and reduce variability among environmental parameters, thereby improving model performance [54]. Such models, developed using regional datasets (e.g., cadmium accumulation in maize cultivated in Kenyan soils), are useful for preliminary assessments of metal uptake. However, they are empirical in nature and do not explicitly account for the underlying physical and geochemical mechanisms governing metal transport and bioavailability. To better characterize metal mobility under field conditions, empirical models should be complemented by kinetic and transport-based approaches. Kinetic models describe the rates of metal release, adsorption, and desorption from soil solid phases, thereby providing insight into contaminant availability over time. For example, the Elovich and parabolic diffusion models have been successfully applied to investigate the kinetics of chromium desorption from Nigerian Vertisols [55]. Unlike simple adsorption isotherms, these models help explain the dynamic interactions between soil minerals, organic matter, and contaminants, making them more relevant for predicting long-term metal mobility and optimizing phytoremediation strategies in the heterogeneous soils commonly encountered across African ecosystems.

### 7.2. Geochemical and Transport Models

Geochemical speciation programs such as Visual MINTEQ and PHREEQC can predict the activities of free metal ions in soil solution from the effect of pH, ionic strength, and other competing cations. For instance, Visual MINTEQ predicted that Cu^2+^ activity in the Zambian Copperbelt soils was regulated by Cu-organic complexes where pH > 6. Such information would help in selecting the appropriate hyperaccumulator as free Cu^2+^ is the available species. HYDRUS-1D models metal transport in the unsaturated zone one-dimensionally. The combined use of geochemical speciation and transport models offers a more realistic assessment of metal mobility and bioavailability than empirical models alone, making them particularly valuable for phytoremediation planning in heterogeneous African soils. In applying the model to assess phytoremediation of lead-contaminated soils in Egypt, HYDRUS estimated the extent of metal leaching within ten years to provide the EDTA application rates [58].

### 7.3. Geospatial and Machine Learning Approaches

Machine learning demonstrates better predictive power for larger datasets; however, its practical application in African environmental systems remains constrained by limited high-quality datasets, inadequate computational infrastructure, inconsistent monitoring frameworks, and insufficient field-scale validation studies. Random Forest (RF), Artificial Neural Networks (ANN), and Support Vector Machines (SVM) have shown promising predictive capability for estimating heavy metal distribution and contamination risks [59]. For example, in Ghana’s Tarkwa mining district, RF predicted soil Hg concentration with an R^2^ of 0.79 using elevation, drainage density, and vegetation index. ANN outperformed linear regression in predicting Cu concentrations in DRC Copperbelt soils, while SVM successfully classified Pb contamination levels in South Africa. Despite these advances, many models remain calibrated using localized datasets with limited transferability across heterogeneous African soil systems. Furthermore, uncertainties associated with remote sensing resolution, data scarcity, and overfitting continue to limit large-scale implementation and policy integration [60]. Machine learning algorithms integrated with GIS-based spatial analysis improve contamination prediction accuracy and facilitate prioritization of remediation sites. Remote sensing technologies and random forest models are increasingly used for large-scale soil contamination assessment [35,57,59].

### 7.4. Model Limitations and Validation

Models are seldom validated on independent African datasets, and most studies rely on calibration and validation using the same dataset, increasing the risk of overfitting. This limitation reduces the reliability and scalability of predictive tools for real-world remediation planning. Proper validation approaches, such as independent dataset partitioning and K-fold cross-validation, are therefore essential. Additionally, models calibrated using temperate-region soil spectra often perform poorly on African Oxisols due to mineralogical and geochemical differences. These findings emphasise the urgent need for region-specific datasets, harmonised monitoring systems, and long-term field validation studies across African environments [61].

## 8. Molecular and Genetic Approaches

### 8.1. Mechanisms of Metal Uptake and Tolerance

Heavy metal contamination in African ecosystems adversely affects soil quality, microbial diversity, and native flora and fauna by disrupting nutrient cycling, reducing plant productivity, and altering rhizosphere microbial communities. Prolonged metal exposure can diminish biodiversity and impair ecosystem resilience, highlighting the need to understand the molecular mechanisms underlying metal tolerance and adaptation in native hyperaccumulator species. Transporter genes include ZIP transporter family (Zn, Cd, Fe transport), NRAMP family (Cd, Mn, Fe transport), HMA (ATPases heavy metal transporters; involved in excretion and vacuolar sequestration), and metal tolerance protein (MTP) [62]. Tolerance mechanisms include phytochelatin chelation (PCs) and metallothionein chelation (MTs), vacuolar sequestration, and antioxidant defense (SOD, CAT, APX).

In “*Berkheya coddii*”, tolerance to Ni occurs through constitutive high expression of the “BcNRAMP” gene and vacuolar sequestration of Ni by the “BcMTP1” gene. African hyperaccumulator plants may have distinct alleles not observed in model plants.

### 8.2. Omics-Based Insights

Transcriptomics, the RNA-seq of “*Haumaniastrum robertii*” growing in Co-enriched soil, showed upregulation of vacuolar Co transporters (HMA3-like) and nicotianamine synthase. Proteomics, “Bidens Pilosa” treated with Cd, showed upregulated glutathione S-transferase and heat shock proteins by proteomics [63]. Metabolomics, Ni hyperaccumulation in “Senecio coronatus” is associated with an increase in histidine and malate, which help chelate Ni in xylem sap.

### 8.3. Breeding and Genetic Engineering

An alternate method that may prove more efficient is genetic engineering. Transgenic plants overexpressing metal transporter genes have shown enhanced metal accumulation capacity under controlled conditions. Recent advances in omics technologies, CRISPR-Cas genome editing, and heavy metal ATPase gene regulation have significantly improved understanding of plant metal transport mechanisms and stress tolerance pathways [60,61,63]. However, the practical application of genetically engineered phytoremediation systems in Africa remains limited by biosafety regulations, high implementation costs, inadequate technical infrastructure, ecological uncertainties, and low public acceptance of genetically modified organisms (GMOs). Although engineering native African grasses such as *Pennisetum purpureum* represents a promising strategy, field-scale feasibility, long-term ecological impacts, and regulatory approval processes require careful evaluation before deployment [64].

### 8.4. Biosafety and Regulatory Considerations

The majority of African states have signed up to the Cartagena Protocol on Biosafety, which requires a risk assessment before any release of GMOs. Nonetheless, only South Africa, Burkina Faso, and Sudan have systems in place to facilitate the release of GMOs. Non-GMO-based techniques that employ native hyperaccumulators or PGPR for phytoremediation would be easier to manage from a regulatory perspective and be more acceptable to the general population [65].

## 9. Integrated Framework, Challenges, and Future Directions for Phytoremediation in Africa

**Figure 2:**
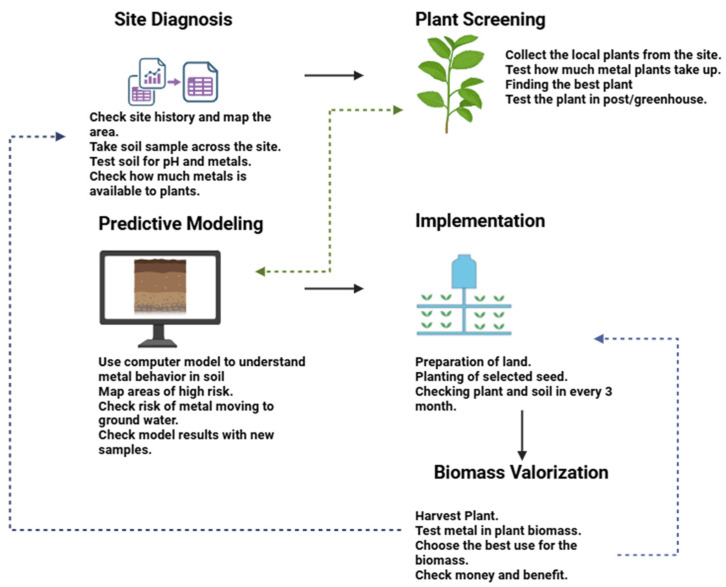
Integrated Framework for Heavy Metal Assessment and Phytoremediation of Contaminated Soils (1) Site diagnosis, including soil sampling, mapping, and assessment of pH and metal availability; (2) Plant screening, where local plant species are collected and evaluated for metal uptake under field or greenhouse conditions; (3) Predictive modeling, using computational tools to understand metal behavior, identify high-risk zones, and validate predictions with new samples; (4) Implementation, involving land preparation, planting of selected species, and periodic monitoring of soil and plant status; and (5) Biomass valuation, where harvested plants are analyzed for metal accumulation and evaluated for safe use and economic benefit. Dashed arrows indicate feedback loops for continuous optimization across stages.

Phytoremediation technology for contaminated African soils will be dependent upon the integration of various technical approaches along with socio-economic factors, with particular emphasis on current deficiencies and future improvements. The initial stage involves site evaluation using methods like pXRF analysis, physicochemical testing and sequential extraction of metals to determine bioavailability [66]. Subsequently, the plant selection process includes the use of greenhouse testing and field trials to screen out indigenous and fast-growing plants with high uptake and translocation abilities. The use of modelling techniques such as geochemical modeling programs and machine learning techniques helps in predicting mobility and variability of metals [67]. In addition to the use of hydrological models for leaching risks, the implementation phase will involve the use of soil amendments such as PGPR, mycorrhizae and biodegradable chelating agents. Finally, biomass management for economic benefits and community engagement is considered shown in Figure 2 [68].

Stakeholder involvement is equally critical, and this involves using a participatory approach to involve stakeholders such as farmers, policymakers, and the local community to improve adoption and sustainability [52]. Economic feasibility is assured by the ability to recover land values, generate carbon credits, receive revenues from phytomining, and lower health care costs. While all these improvements have potential benefits, various hurdles still exist, such as limited validation in the field, incomplete identification of hyperaccumulating plants within an area, lack of knowledge about mixed contaminants, and inadequate laboratory regulations [69]. Circular phytomining and integration with renewable energy sources are potential economic models for consideration, while the need for harmonized biosafety frameworks will be critical for developing gene-edited plants. Ultimately, capacity development remains key to success in phytoremediation endeavors on the continent [70].

Despite significant advances, the large-scale implementation of phytoremediation in Africa remains constrained by limited field validation, inadequate research infrastructure, insufficient identification of native hyperaccumulator species, and socioeconomic and regulatory challenges. Future progress will require stronger environmental policies, increased investment in research, and multidisciplinary collaboration. Although plants with BAF and TF values greater than 1 are considered suitable for phytoextraction, effective remediation also depends on high biomass production, robust root systems, climatic adaptability, and beneficial rhizosphere interactions [44,47,48,49,62,64].

## 10. Conclusions

Metal pollution of African soils is not only a consequence of industrial activities, mining operations, rapid urbanization, agricultural intensification, and regulatory shortcomings, but also reflects the influence of diverse African-specific environmental, climatic, agricultural, and socioeconomic conditions that affect contaminant distribution and remediation efficiency. However, this challenge also presents an opportunity for sustainable green technologies. The utilization of Africa’s diverse native hyperaccumulator plant species, combined with emerging advances in predictive modeling, molecular biology, and genetic approaches, offers promising strategies for soil restoration and recovery of economically valuable metals. In this revised framework, additional comparative insights regarding regional contamination patterns, native hyperaccumulator diversity, and practical implementation barriers across African countries have been incorporated to improve contextual relevance and scientific depth. Nevertheless, successful implementation will require transitioning from small-scale experimental studies to economically feasible, large-scale field applications supported by stronger infrastructure, policy development, and environmental monitoring systems. Soil is a finite and vulnerable resource, and its restoration remains an urgent environmental priority.

## Figures and Tables

**Table 1:** Priority Heavy Metals in African Soils—Sources, Thresholds, and Health Effects.

Metal/Metalloid	Primary Anthropogenic Sources in Africa	Typical Background (mg/kg)	Contaminated Soil Range (mg/kg)	Phytoremediation Mechanism	Human Health Effect (Chronic Exposure)	References
Lead (Pb)	Artisanal mining, battery recycling, leaded petrol (legacy), paint	10–30	150–16,000+	Phytostabilization (preferred); weak phytoextraction	Neurodevelopmental toxicity (children); nephrotoxicity; hypertension	[15]
Cadmium (Cd)	Phosphate fertilizers, sewage sludge, Zn mining	0.1–0.5	0.14–6.07	Phytoextraction (highly feasible)	Itai-itai disease; kidney tubular damage; bone demineralization	[16]
Mercury (Hg)	Artisanal gold mining (amalgam burning), chlor-alkali plants	0.01–0.1	0.51–1830	Phytovolatilization (by engineered plants); rhizofiltration	Neurotoxicity; tremors; teratogenicity	[17]
Arsenic (As)	Gold mine tailings, volcanic geogenic, pesticide legacy	1–10	0.1–399 (in vegetables)	Phytoaccumulation (e.g., *Pteris vittata*); phytostabilization	Skin lesions; bladder/lung cancer (Group 1 carcinogen)	[18]
Chromium (Cr)—Cr(VI)	Tanneries, electroplating, chrome mining (South Africa)	20–100 (total)	11.9–119.3; up to 4000 in tanneries	Reduction to Cr(III) + phytostabilization; phytoextraction (low)	Lung cancer; skin ulcers; respiratory distress	[19]
Zinc (Zn)	Zn mining, galvanized waste, manure	20–50	38–1454	Phytoextraction (very feasible; many hyperaccumulators)	Stomach cramps; anemia (at very high doses)	[20]

**Table 2:** Comparison of Analytical Techniques for Heavy Metal Characterization in African Soils.

Technique	Metal(s) Detected	Detection Limit (mg/kg)	Advantages in the African Context	Limitations	References
Portable XRF (pXRF)	Pb, Cu, Zn, As, Cr, Ni, Co, Fe	10–20 (varies by element)	Rapid screening; non-destructive; low consumable cost; used successfully in Ghana	Poor for Hg; matrix effects (moisture, organic matter)	[43]
Flame AAS (FAAS)	Pb, Cd, Cu, Zn, Ni, Cr, Co, Mn	0.5–2	Widely available in African labs; robust; validated in Ethiopia, Nigeria, Kenya	Requires digestion; single-element analysis; not for As/Hg	[44]
BCR Sequential Extraction	Operationally defined fractions (exchangeable, reducible, oxidizable, residual)	Same as detection method	Predicts bioavailability; informs remediation choice; used in Nigeria	Time-consuming (3–4 days); not standardized across labs	[45]

## Data Availability

No new data were created or analyzed in this study.
